# WHO public health laboratories webinar series – an online platform to disseminate testing recommendations and best practices during health emergencies

**DOI:** 10.3389/fpubh.2024.1462756

**Published:** 2025-01-15

**Authors:** Céline Barnadas, Lisa Stevens, Natacha Milhano, Ana Carolina Barbosa de Lima, Bruce Struminger, Lauren Burke, Sébastien Cognat

**Affiliations:** ^1^Public Health Laboratory Strengthening Unit, WHO Lyon Office, Health Emergencies Programme, World Health Organization, Lyon, France; ^2^Independent Consultant, Lisbon, Portugal; ^3^ECHO Institute, University of New Mexico Health Sciences Center, Albuquerque, NM, United States

**Keywords:** learning, laboratories, health, emergencies, epidemics, disease outbreaks, public health

## Abstract

Laboratories play a central role in managing public health emergencies. The COVID-19 pandemic imposed unique challenges on global laboratory systems, including testing protocol uncertainties, supply shortages, rapid need for information dissemination, and disruptions to traditional training methods. In response, the WHO established the Public Health Laboratories (PHL) knowledge sharing webinar series whose goals were to respond to the increased demand in up-to-date and reliable information, which WHO is in a unique position to provide. It also aimed to enhance peer-to-peer exchanges across laboratories. This article outlines the PHL webinar series delivery format and presents how the webinar series was received and perceived by participants and how it evolved to support the response to other health emergencies. Contents of the knowledge sharing sessions, as well as attendance, participants’ satisfaction and application of learning were monitored over time using registration forms, satisfaction polls, an annual survey and focus group discussions. From May 2020 to December 2023, 48 sessions attracted 58,688 registrations from 204 countries and territories. Thirty-five sessions featured presentations of WHO guidance, tools or documents and 39 sessions featured country experience sharing. Initially focused on COVID-19, the series became a tool to rapidly disseminate guidance and best practices during new health emergencies and to address cross-cutting topics relevant to the laboratory workforce. Feedback data shows participants found the webinars very useful (86% respondents), reporting knowledge gains in biosafety, quality management, and laboratory practices. The series facilitated knowledge application, with foreseen changes in workplace procedures and training activities (43% respondents). Barriers such as resource limitations, additional training needs, and connectivity issues were frequently identified. Evidence that this knowledge was subsequently applied by participants, such as through changes in workflow, onwards training events and procedural changes further reinforces the efficacy with which the series was able support the laboratory workforce globally in addressing challenges of the COVID-19 pandemic and other health emergencies. The series utilized sessions on cross-cutting topics to run routinely and to keep a high level of engagement with laboratory professionals globally. This enabled it to act as an adaptable tool that was leveraged effectively and quickly during health emergencies for just-in-time learning.

## Introduction

Laboratories play a central role in public health emergency management, delivering diagnostic services that guide the appropriate clinical management of patients, enable disease surveillance, including detection and confirmation of communicable disease outbreaks, and provide data to inform public health interventions. However, the delivery of timely and appropriate laboratory services of high quality is dependent on various factors specific to a country’s context including resource availability (i.e., testing protocols, supplies, infrastructure, trained workforce) and scientific evidence (i.e., who should be tested, when, and how) and can be impacted by the nature of each emergency (1, 2).

In the context of the COVID-19 pandemic, laboratory systems globally faced an unprecedented number of new challenges in the delivery of laboratory services for this unprecedented and rapidly evolving public health emergency (1). Key questions such as who (i.e., asymptomatic versus asymptomatic) and how to test (molecular versus serological tests, protocols, biosafety measures, etc.) were problematic due to an initial lack of information about disease transmission, and an influx of new diagnostic tests being brought to market with sometimes limited performance data (3). Challenges such as supply shortages, personnel shortages, and fear among health and laboratory workers added to a critical need for timely information dissemination and exchange which was further disrupted by travel restrictions and border closures preventing traditional face-to-face training and laboratory visits (4).

The use of virtual learning has significantly increased in recent years with development of communication technologies and has been widely applied for health topics, such as through the ECHO model (5) established in 2003 to strengthen health workers continuing professional development and capacity building.

It was in this health emergency context and leveraging the increasing digital learning environment opportunities that the Public Health Laboratories (PHL) webinar series was established by the Public Health Laboratory Strengthening Unit at WHO Headquarters (Lyon office), the WHO Regional Office for Africa (AFRO), and the WHO Regional Office for the Eastern Mediterranean (EMRO). Initially designed to support national reference laboratories performing SARS-CoV-2 testing across the African (AFR) and Eastern Mediterranean (EMR) regions, the webinar series was developed with the goals of enhancing WHO guidance and best practices dissemination by communicating with key laboratory stakeholders at country level and enhancing knowledge sharing and peer-to-peer exchanges across laboratories in these regions. In line with the COVID-19 health emergency, it prioritized topics to support laboratories in establishing and scaling up quality and timely testing in a safe manner. However, the audience quickly grew as the series gained interest and support from all WHO regional offices and became relevant to many subnational laboratories and other global laboratory stakeholders. In an effort to strengthen the monitoring and evaluation of the series, WHO engaged with Project ECHO at the University of New Mexico, an initiative established in 2003 to strengthen health worker continuing professional development and capacity building through an “all teach, all learn” tele-mentoring approach (6–8).

In this article we outline the PHL webinar series delivery format and present how the webinar series was received and perceived by participants contributing to the increasing evidence on the added value of e-learning platforms for continuous education and just-on-time learning in health emergencies. We highlight that the series not only met its initial goals to rapidly disseminate information and encourage knowledge exchange among WHO Member States during the COVID-19 pandemic, but also established an enduring channel for multidirectional communication and learning between WHO offices, Member States, and other global laboratory stakeholders. Its sustained relevance demonstrates its potential as a valuable resource for future health emergencies.

## Materials and methods

Ninety-minute webinar sessions were conducted approximately every 2 to 4 weeks. Learning sessions typically included presentations by speakers from WHO headquarters or regional offices on featured WHO guidance, followed by experience sharing from invited speakers from one to three countries. Webinars were delivered primarily in English, with occasional country presentations made in French or Spanish. Live interpretation was provided with several languages added gradually to ultimately offer six languages: English, French, Spanish, Portuguese, Russian and Arabic. At the end of each webinar a designated questions and answers (Q&A) period allowed questions from participants to be relayed to speakers. Additional elements were sometimes integrated such as technical polling questions to enhance interactions with the participants and as an information gathering tool for WHO.

Invitations were disseminated within regional networks, by independent sharing between colleagues (“word of mouth”) and eventually through a registrant database whereby new invitations would be sent to all registrants of previous sessions. A one-page summary covering the highlights of the event, links to the Q&A, recordings of the session, speakers’ PowerPoint presentations, and other relevant documentation were emailed to participants, generally in the week following the webinar. This document was made available in English and French for most sessions from 2021 onwards, with a version in Russian being added in the second half of 2022.

### Data collection

All data collection tools utilized in this study are provided as Supplementary materials. Data was collected from session agendas and Zoom registration forms. Additional monitoring and evaluation tools were also applied including satisfaction polls, an annual survey, and focus group discussions to provide additional user feedback (Table 1). Information on the frequency and timeframe with which these tools were applied is presented in Table 1.

**Table 1:** Data collection tools frequency and timeframe.

Data collection tool	Frequency	Timeframe
Session agenda	Every session	May 2020 – December 2023
Registration	Every session	May 2020 – December 2023
Satisfaction polls	14 sessions	June 2022 – December 2023
Annual survey	Once	September 2021 – September 2023 (covering 22 sessions)
Focus groups	Once (1 in English and 1 in French)	September 2021 – December 2023

Zoom registration enabled the collection of demographics for the registrants of each session and contained the registrants’ name, email, city, country, gender, preferred language (among those offered), profession/role, health system level of work, suggestions, and any pressing questions (Supplementary material 1). Of note, registrant affiliation data (profession/role and health system level of work) only started being collected through the registration form from February 2022 onwards (Session 28).

Satisfaction polls started in June 2022 (session 33) and included four questions about session relevance to the participant’s work, the usefulness of examples and information shared, commitments to taking action based on session learnings, and the likelihood of recommending the learning series to a colleague (Supplementary material 2). The satisfaction poll was launched in the last few minutes of the online webinar sessions, in English, with live simultaneous interpretation into Arabic, French, Portuguese, Russian, and Spanish.

An annual survey including 13 questions solicited feedback about attendance, barriers to participation, useful webinar elements, knowledge acquired, knowledge application and barriers, and the likelihood of recommending the series to a colleague (Supplementary material 3). This data was collected and managed using REDCap (Research Electronic Data Capture), a secure web-based software platform designed to support data capture for research studies (9). In November 2022, an invitation email containing a link to the survey was sent to anyone who had registered for one or more of the PHL webinar sessions since September 2021. The survey was made available in Arabic, English, French, Portuguese, Russian and Spanish.

On the annual survey, respondents were asked if they would agree to be contacted to provide further feedback about the webinars in a more workshop-style setting. A sample of those who responded positively was invited by email to participate in a virtual (Zoom) focus group discussion – the sample considered the gender and region of respondents to maintain general attendance diversity and representativeness. One discussion was conducted in English, and a second discussion was held in French. Discussions followed a semi-structured guide with questions and prompts focused on session improvements and practice change (Supplementary material 4).

### Data analysis

Frequencies and other descriptive statistics were calculated for annual survey variables as appropriate using R (10) and RStudio (11). Focus group discussions were recorded, transcribed, and systematically analyzed using principles of content analysis to identify key concepts (12). Transcripts were analyzed in their original language (English or French), and example quotes presented in the findings section were translated into English when necessary. NVivo software was used for qualitative analysis of focus group transcripts and open-ended recommendations captured in the annual survey (13).

## Results

### Summary of sessions

Between May 2020 and December 2023, a total of 48 webinar sessions were conducted. A cumulative 58,688 registrations were received from 204 countries and territories, with 24,261 attendances. The median attendance was 504, ranging from 91 to 1,558 participants. Annual median attendance was 185 in 2020, 655 in 2021, 507 in 2022 and 588 in 2023. The top three most attended sessions were those on mpox viral disease (*n* = 1,558 participants), Laboratory Management/General management processes (*n* = 1,238 participants) and Biosafety Risk Assessment tools and processes (*n* = 1,124 participants).

The two primary forms of knowledge-sharing and information dissemination used were presentations of WHO guidance, tools or documents (35 sessions) and country experience sharing (39 sessions). In each session featuring country experience sharing, panelists from one to three countries were invited to participate. In total, panelists from 45 countries presented during at least one session. All WHO regions (17) were represented with experience sharing from 16 countries from the African region, six for the Eastern Mediterranean region, 11 from the European region, five from the region of the Americas, three from the South-East Asian region and four from the Western Pacific region. Presentations by countries were primarily given in English, however three presentations were delivered in French and two in Spanish.

**Figure 1:**
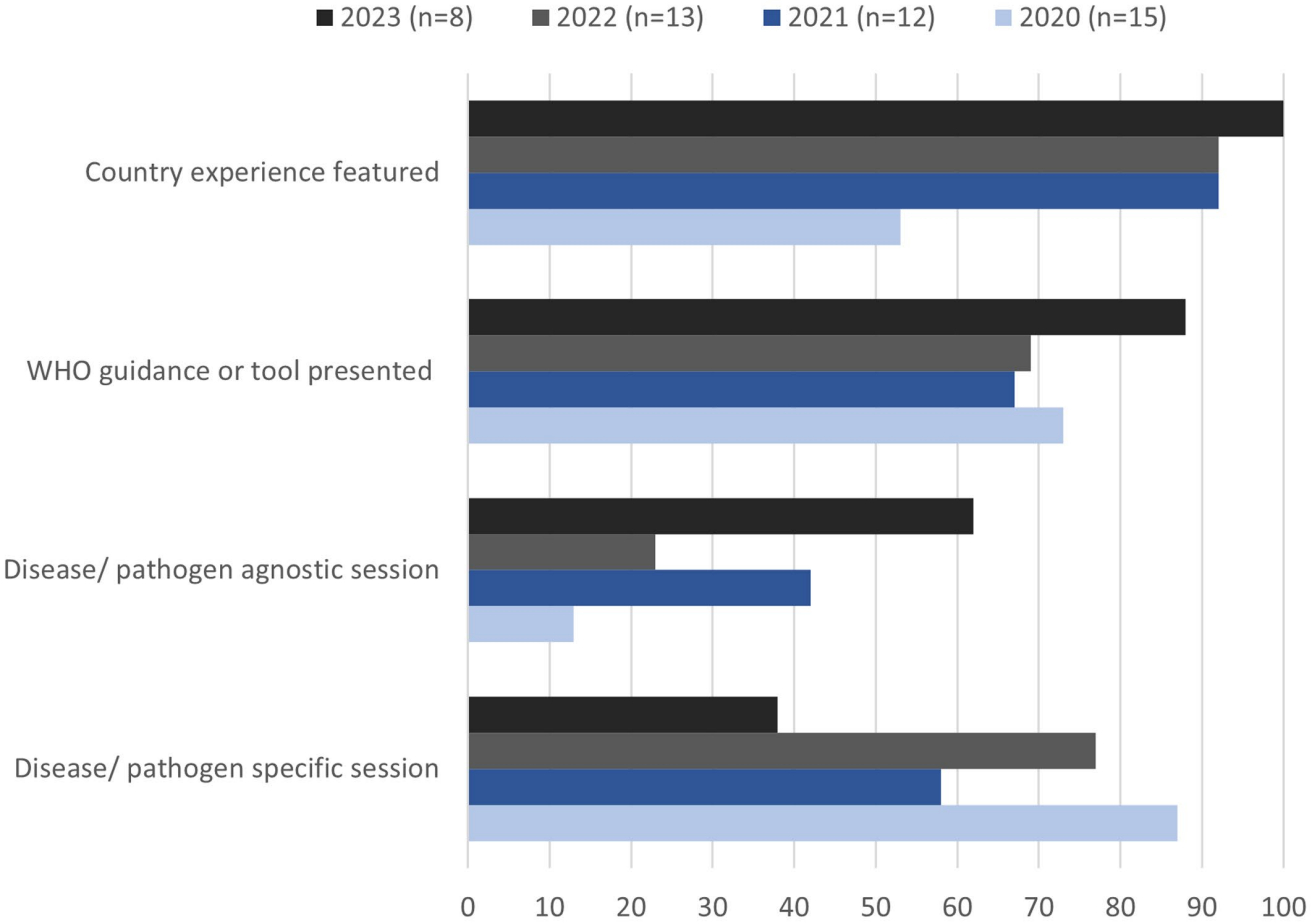
Main contents of the PHL webinar series, in proportions of all sessions run annually from 2020 to 2023.

Most of the sessions delivered in the first 2 years of the series related to SARS-CoV-2 diagnosis (24/32), with 67% over the entirety of the webinar sessions (32/48) dedicated to the disease (Figure 1). From September 2021 onwards, cross-cutting pathogen-agnostic sessions began and included topics such as biosafety, laboratory and emergency management processes, quality management systems and communication for laboratory stakeholders.

Additional diversification of topics included pathogen-specific sessions on diseases other than COVID-19. This included seven webinars on diagnostics for specific diseases including mpox, Sudan virus disease, HIV, acute hepatitis, leptospirosis, and diphtheria. During these sessions information delivered included raising awareness of new WHO guidance publications, epidemiological updates of recent outbreaks, recommendations on best-practice testing methods, the use of new or innovative technologies, or regulatory requirements.

### Registrant profiles

From the 33 webinars for which this information was available (March 2021–December 2023, *N* = 52,494), 62% of registrants were female, 36% male, 1% preferred not to say, and 0.1% identified as non-binary. Fifty-six percent of individuals (*n* = 19,656) registered for more than one webinar, with one individual registering for 37 sessions. Moreover, from the 560 respondents to the 2022 annual survey, 65% of respondents reported attending between one and five sessions, 25% attended between six and 10 sessions and 11% more than 10 sessions.

Since February 2022 (session 28), demographic data from 35,827 registrants indicated that 47% described their role as laboratory personnel, 11% as researcher, 8% as technical officer, 7% as a public health official, 7% as consultant, 6% as program manager, 4% as medical care provider, 3% as student and 7% as other. Of those who answered the optional question about the level of health system in which they worked (*N* = 26,080), the majority (55%) reported working at national level, 26% at subnational level, 12% at other levels such as in international organizations and/or humanitarian organizations and for 8% the question was left blank or marked non-applicable.

The majority of registrants were from the Western Pacific region (29%), followed by the African region (21%), European region (20%), South-East Asia region (15%), Eastern Mediterranean region (10%) and the Region of the Americas (6%). Overall, 204 countries and territories were represented among participants, with the five most represented countries being Philippines, Indonesia, Ukraine, Nigeria and India.

### Reception and perception of the series by participants

From satisfaction polls’ respondents (*N* = 3,126), over 80% indicated that the webinars were either very relevant or extremely relevant to their work. In terms of usefulness, most respondents rated the webinars as being very useful (86%), and less than 1% of respondents stating they had not been useful to them. During focus group discussions, nine out of 16 focus group participants mentioned interest and relevance of a particular topic to their work being the primary motivation for joining the webinars with one participant mentioning that the topics were critical for her as a trained biologist working at a national public health laboratory.

**Figure 2:**
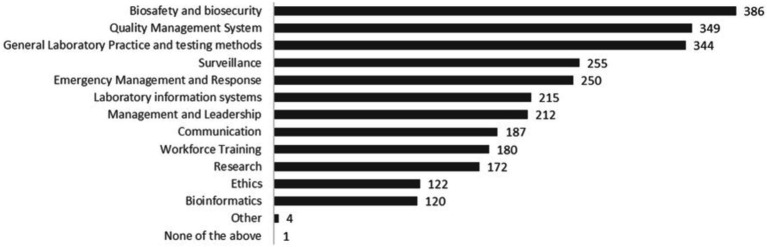
Areas of new knowledge learnings or skills for participants (annual survey, *N* = 560 respondents).

Annual survey respondents (*N* = 560) indicated a wide range of areas in which they gained new knowledge or skills, with the top selected areas (indicated by more than 60% of respondents) including biosafety and biosecurity, quality management systems, and general laboratory practice and testing methods. Other popular areas of learning (indicated by more than 30% of respondents) included surveillance, emergency management and response, laboratory information systems, management and leadership, communication, workforce training, or research (Figure 2).

According to annual survey respondents, the most helpful elements of the webinar series were learning the latest technical developments (83% rated it as very helpful, *n* = 466) and hearing experiences from other countries (81% rated it as very helpful, *n* = 453). Links to WHO documents and learning from peers were also among the topmost helpful elements, selected as very helpful by 75% (*n* = 420) and 73% (*n* = 408) of respondents, respectively. In focus group discussions, session content that was cross-cutting and pathogen-agnostic was identified among the most helpful, including biosafety, biosecurity and biological risk assessments (4 of 16 participants), laboratory management skills (3 participants) and leadership and communication skills (one participant). Two participants also highlighted sessions featuring guidance on specific disease testing as the most helpful for them.

In terms of likelihood of recommending the webinars to colleagues, using a Likert scale of 1- not at all likely to 10- extremely likely, 83% of respondents in session satisfaction polls (*n* = 2,977) rated their answer as equal or higher than 8. The annual survey also showed a high likelihood of recommending the webinar to a colleague, with more than 50% of respondents (*n* = 290) selecting 10, extremely likely to recommend, and 87% (*n* = 481) of respondents’ rating 8 or above.

### Knowledge application and best-practice implementation

**Figure 3:**
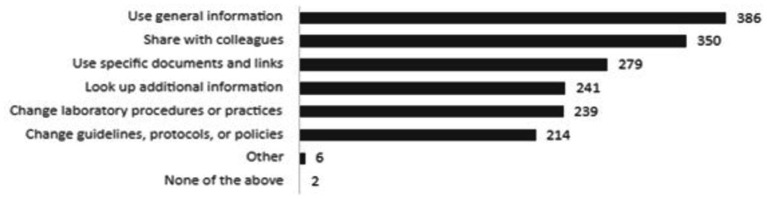
Ways participants planned to use learnings (annual survey, *N* = 560).

**Figure 4:**
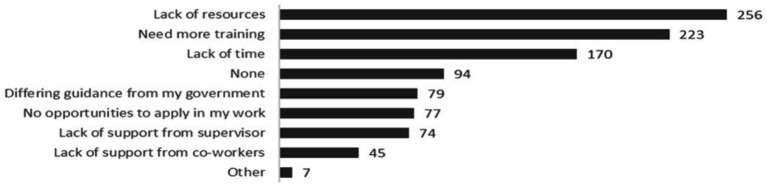
Knowledge application barriers (annual survey, *N* = 560).

In satisfaction polls (*N* = 3,126), participants indicated they intended to apply the knowledge or products obtained from the webinar series by sharing information or products with colleagues (32%), looking up additional information (25%), improving a process in their workplace (24%) or improving the way that they worked (17%). During the annual survey, respondents also mentioned the intention to use information to change laboratory procedures and practices (43% of respondents) or guidelines, protocols or policies (38% of respondents) (Figure 3). In focus groups, concrete examples were shared of how the best-practices were applied in practice (Table 2). In the annual survey (*N* = 560), challenges to applying knowledge or implementing the best-practices shared were also identified, with the top three barriers including a lack of resources (46%), need for more specific training (40%), and lack of time (30%). Other less common barriers included a misalignment between best practices shared and the guidance provided from the government, no opportunities for application, and lack of support from supervisors or co-workers (Figure 4).

**Table 2:** Knowledge application themes from focus groups (16 participants).

Knowledge application*	Number of participants	Number of quotes	Example quote
Implemented procedures based on risk assessment	4	10	“We were worried about how the RDT test can be managed. (…) after the training organized by the WHO, (…) we have learned about the RDT management. It can be done in a simple way. So I think it also decreased our burden.”
Increased awareness and understanding of potential risks	3	4	“We encountered many suspected cases of monkeypox. Although it was negative, but the webinar sessions kept me informed and aware about this outbreak.”
Training	3	4	“(…) what I was able to change after attending this webinar series. (…) among the trainings I conducted during the pandemic, I included IPC measures, quality assurance, and quality control measures.”
Shared with colleagues	3	4	“All sessions were very informative, and I have also shared my experience with my staff.”
Improved laboratory management skills	3	3	“I had tried autocratic management, where (…) I decide everything, and after the training, I discovered that (…) each management style must be adapted according to the context. (…) it allowed me to take into account what the staff says (…) the staff is satisfied with the approach I have toward them.”
Informed national protocols	2	2	“These series have brought me a lot, a lot of help, even with advice to the Ministry of Public Health of our country so that there is an elaboration of a national grouping on biosafety”
Adapted for other contexts	1	1	“One of the series I enjoyed so much was about SARS-Cov-2, (…) it has not only helped us in COVID-19 management of SARS-CoV-2 (…) the information we learned some time back is being helpful even right now where we are battling with Ebola.”

*Supplementary material 5 outlines the definitions of knowledge application themes.

During the focus groups (*N* = 16), participants shared additionally that challenges related to supply chain logistics prevented best-practice implementation. For example, one participant said: “We wanted to (…) make the tests faster during COVID. We procured the [reagent] dispenser, but (…) procurement and delivery took so long that when we got it, there was not so many new cases anymore.” Two participants shared that the cost of implementation of certain practices was a barrier. Other issues mentioned by a single participant were lack of decision-making power, lack of enforcement from different levels, a need to reinforce awareness, and general resistance to change.

### Engagement with the webinar series

In the annual survey (*N* = 560), the greatest challenges reported in engaging with the sessions were the time of day in which they were held (56%) and internet connection issues (31%). Other less common obstacles were topic irrelevance (15%), language availability (7%), length of session (7%), log-in logistics (5%), and software issues (3%). However, 84% of respondents reported watching one or more session recordings.

Out of 560 total respondents, 137 shared recommendations for improving engagement with the webinar series (Table 3). The majority of suggestions related to changes in session logistics (69 mentions). Other recommendations included addressing language issues, for example through subtitle use (10 mentions), improving accessibility of finding webinar products online like recordings (five mentions), having longer sessions (three mentions) and having more time for discussion (three mentions). Outside of logistical concerns, recommendations included proposals of activities complementary to the webinar sessions (29 suggestions), including activities that were practical and hands-on (11 mentions) or involved face-to-face events/trainings (six mentions). Respondents also frequently provided suggestions of future webinar topics they would like to engage with (28 suggestions) with laboratory management and training themes most frequently mentioned (nine mentions).

**Table 3:** Recommendations for improved session engagement (137 annual survey respondents).

Area for improvement	Number of mentions	Example suggestion
Logistics (session time, language issues, recordings accessibility, and others)	69	“Scheduled time is mid-day and difficult to reserve when also often other work-related activities are scheduled at the same time. Maybe several sessions for different time zones during beginning or end of work day.” “English subtitles because sometimes I wish to understand the speakers more but I cannot because of the different accent.”
Develop complimentary activities	29	“Inviting participants for hands on practical” “There is need to set up an award for participants by organizing a virtual expo where participants can showcase what they have acquired or learnt from the webinar series. This will encourage as many people to participate as possible, consequently leading to knowledge spread far and wide.”
Future topics requests	28	“Molecular techniques in details especially RT-PCR and the how to validate regents using different type of technique.” “More topics on laboratory quality control, such as preparation of primers, optional time for each section”
Certificate requests	15	“Most people need certificates of participation”
More guidance or reference materials	12	“Providing more technical documents that are accessible”
Implement knowledge assessments	8	“It might be good to have a post-test after the webinar to make it more effective”
Engage specific stakeholders	3	“Include some speakers from different countries, like Asian countries

In focus group discussions (*N* = 16), nine participants identified similar recommendations for improving webinar engagement with the most common suggestions mentioning changing the session time. However, given the diversity in time zones, the discussion quickly evolved to participants agreeing and acknowledging time zones for a program with a global reach is a great challenge “The time issue is a little bit complicated. So, my other friend in Nigeria wants it at 8:00. In my country, it will be around 5:00. Another wants it at night. (…) So I think it’s a commitment.”

## Discussion

The results of this study add to a growing body of evidence indicating that accessible, learner-centric virtual learning on a global scale can alleviate severe health worker shortages and improve access to updated guidelines and best practices (20, 21). The WHO PHL webinar series provides a unique example of how such a virtual learning model could be leveraged for the education of thousands of laboratory workers and related personnel, providing rapid capacity building and technical support to improve laboratory quality, safety and reliability, particularly in the response to the COVID-19 pandemic.

The rapid growth and expansion of the WHO PHL webinar series in the first year of the COVID-19 pandemic, disseminated through regional networks of webinar invitations and subsequently word of mouth, highlights the demand for such a broadly accessible knowledge sharing platform. The breadth of session topics and presentations and diverse global participation demonstrates the extraordinary reach of the PHL webinar series. It exemplifies the unique convening power of WHO in bringing together global subject-matter experts in a timely manner to enhance the dissemination of WHO guidance and laboratory best practices for a wide range of diseases to a cross- disciplinary audience. This was facilitated by a webinar format that prioritized accessibility and inclusivity, featuring case presentations from 45 countries including 22 from low- and lower-middle-income countries and interpretation into various languages. Additionally, session recordings, summaries, Q&As and links to other relevant materials provided opportunities for laboratory professionals and other health workers facing issues like connectivity and timing challenges to access the learning opportunities of the initiative.

Participant feedback through satisfaction polls, annual survey and focus groups provided insight into the webinars’ effectiveness at enhancing knowledge-sharing specific to laboratory personnel, which represented almost half of all participants. High relevance ratings and repeated indication that participants would recommend the series to colleagues demonstrated that the PHL webinar series’ addressed the professional needs of the public health laboratory workforce. This relevance was enhanced by mechanisms allowing participants to ask questions ahead of and during each session, ensuring participants concerns and knowledge gaps were addressed and enabling suggestions for future session topics. This flexibility ensured just-in-time learning, responding to emerging needs such as new SARS-CoV-2 variants and the need to monitor their spread to inform public health recommendations and interventions or address knowledge gaps expressed by participants such as risk management for SARS-CoV-2 testing, which in turn triggered dedicated biosafety sessions, among others. Evidence that this knowledge was subsequently applied by participants, such as through changes in workflow, onwards training events and procedural changes further reinforces the efficacy with which the series was able to make tangible impact at the laboratory level.

While the webinar series was successful in achieving relevant knowledge dissemination, application and exchange, persistent barriers toward implementing changes were also highlighted. These included insufficient resources and time, the need for more workplace support, and additional training requirements. Connectivity issues also posed participation challenges, common in underserved areas (14–16). Importantly, while the series proved effective at disseminating best practices broadly, participant feedback suggested a continued need for in-person training which stresses that such a series does not replace face-to-face and hands-on learning for laboratory professionals in the technical environment. The series delivery model, with short sessions, large attendance, and simultaneous interpretation, limits interactions among participants, restricting them to text exchange over the chat function of the webinar platform, and limiting the delivery of sessions focusing on problem solving (eg. PCR results interpretation and troubleshooting). These findings provide essential information for WHO to be able to develop future activities to reduce knowledge gaps and better support the laboratory workforce in truly effective, practical implementation of best practices. Recommendations from webinar participants have prompted both WHO and UNM ECHO to reflect on the format of delivery of their webinar series and consider future adaptations to further increase accessibility of the information shared. Additional barriers that were reported such as the lack of resources to implement change continues to be addressed through capacity strengthening initiatives of WHO and other partners, through the national action plans for health security and International Health Regulations (IHR) capacity strengthening (17, 18), the Pandemic Fund initiative which has identified surveillance, laboratory systems and health workforce as its three programmatic priorities (19) and advocacy efforts for stronger laboratories and laboratory systems at the national and subnational level.

A critical limitation of this study is inconsistent data collection at the outset of the series and the low response rate to the annual survey, impeding the ability to generalize findings. Results from different data collection instruments may be biased toward recurring participants during those timeframes. Nevertheless, quantitative and qualitative results show compelling evidence of knowledge acquisition, practice change, and session satisfaction within the study sample. It also provides insights on potential improvements as the webinar continues to be implemented.

Finally, the WHO webinar series successfully integrated topics beyond COVID-19, addressing knowledge gaps for other high priority diseases and cross-cutting laboratory topics. Subjects like laboratory biosafety or quality management that would previously have been delivered using face-to-face trainings could now rapidly reach a broader and more diverse audience through the webinar series. For example, the webinar session on biosafety (session 26) engaged 1,124 individuals from 118 countries. The series also supported emergency responses, such as the Public Health Emergency of International Concern (PHEIC) for mpox in July 2022, with a dedicated session attended by over 1,500 participants from 145 countries, one of the most attended sessions. Similar sessions followed outbreaks of Sudan virus disease (Uganda, November 2022), acute viral hepatitis (June 2022), and diphtheria (Nigeria, July 2023), proving the series to be a flexible tool for WHO to address long-term capacity building and support emergency response efforts.

In conclusion, the WHO Public Health Laboratories’ webinar series successfully achieved its primary goals, which included amplifying the dissemination of WHO guidance and best practices by engaging with key laboratory stakeholders worldwide. It aimed to bolster knowledge sharing, facilitate peer-to-peer interactions among laboratories, and enrich WHO’s insights into the prevailing knowledge deficits and obstacles to implementing guidance and best practices.

The webinar series succeeded in not only supporting the laboratory workforce in addressing challenges of the COVID-19 pandemic but was also successfully leveraged to support learning on cross-cutting laboratory topics and outbreak response for other epidemic and pandemic prone diseases. This success was possible because the series was implemented continuously, alternating cross-cutting topics and rapidly addressing new health emergencies. This approach allowed for the reactivation of an essential and relevant contingent of the global laboratory workforce in a timely manner, facilitating just-in-time learning for a more effective response to regional and global health emergencies. A similar approach may be utilized by WHO and technical agencies in other areas of work related to health emergencies.

Implementing stronger monitoring and evaluation mechanisms was essential for assessing the quality and delivery of the webinar series and its usefulness for participants. Evaluating its impact is crucial to justify resource allocation and drive continuous improvement, leading to greater impact and more effective support for the laboratory workforce and stakeholders who provide essential health services globally. Through the ongoing PHL webinar series, WHO will continue to utilize its unique convening power, inviting world class laboratory and diagnostic experts to help improve access to, and dissemination of, quality guidance and best-practices for the laboratory workforce globally.

## Data Availability

The datasets presented in this article are not readily available because requests to access data will be evaluated according to the ethics guidelines and should be directed to the corresponding author. Requests to access the datasets should be directed to Céline Barnadas, barnadasc@who.int.
