# Supplementary material for: WHO public health laboratories webinar series – an online platform to disseminate testing recommendations and best practices during health emergencies
Source: Front Public Health. 2025 Jan 15;12:1462756. doi: 10.3389/fpubh.2024.1462756 (PMC11775005; doi:10.3389/fpubh.2024.1462756)
Supplement: Supplementary file 1 [file Table_1.docx]

Supplementary Material

**Supplementary Material 2. Satisfaction poll**

1. How relevant was this session to your current work?

❑ Not at all relevant

❑ Slightly relevant

❑ Moderately relevant

❑ Very relevant

❑ Extremely relevant

1. How useful were examples or information shared in this session?

❑ Not very useful

❑ Somewhat useful

❑ Very useful

❑ Not applicable – nothing was shared

1. Leaving this webinar, which actions or challenges would you like to commit to tackling? Check all that apply

❑ Improve how I work

❑ Improve a process in my workplace (guidelines, SOPs, etc.)

❑ Share what I learned or webinar products with colleagues

❑ Look up additional information

❑ Other

❑ None of the above

1. How likely are you to recommend this webinar series to a colleague?

| Not at all  likely | |  | |  | |  | |  | |  | |  | | Extremely  likely | | |
| --- | --- | --- | --- | --- | --- | --- | --- | --- | --- | --- | --- | --- | --- | --- | --- | --- |
| 1 | 2 | | 3 | | 4 | | 5 | | 6 | | 7 | | 8 | | 9 | 10 |
