# Supplementary material for: WHO public health laboratories webinar series – an online platform to disseminate testing recommendations and best practices during health emergencies
Source: Front Public Health. 2025 Jan 15;12:1462756. doi: 10.3389/fpubh.2024.1462756 (PMC11775005; doi:10.3389/fpubh.2024.1462756)
Supplement: Supplementary file 2 [file Table_2.docx]

Supplementary Material

**Supplementary Material 1. Registration form**

First name* 
Last name* 
Email address* 
Confirm email address 
City* 
Country/Region* [dropdown with WHO country options] 
Organization name* 

What gender do you identify as? * 
❑ Male 
❑ Female 
❑ Non-binary 
❑ Prefer not to say 

Webinar language preference:* 
❑ Arabic 
❑ English 
❑ French 
❑ Portuguese 
❑ Russian 
❑ Spanish 
❑ Other (please specify in ‘additional information’ box below) 

Organization type:* 
❑ National reference laboratory 
❑ National Health Institute 
❑ Diagnostic laboratory (public or private sector) 
❑ Academic laboratory or institution 
❑ Hospital or nursing/care facility 
❑ Ministry of Health 
❑ Other ministries 
❑ Non-governmental organization 
❑ United Nations

❑ Other (please specify in ‘additional information’ box below) 

Your role: *^#^
❑ Laboratory personnel 
❑ Medical care provider 
❑ Programme manager 
❑ Technical officer 
❑ Consultant 
❑ Public health official 
❑ Researcher 
❑ Student 
❑ Other (Please describe in ‘additional information’ box below) 

At what level of the health system do you work ? ^#^
❑ National level 
❑ Subnational level (provincial, district) 
❑ Other 
❑ Non applicable 

Do you have suggestions for topics, learning format, etc.? 

Do you want to receive emails from Project ECHO®? Sign up to receive session materials, information on upcoming sessions, learning opportunities, and special events. 
❑ Yes 
❑ No

By submitting this registration form, you hereby confirm your consent to the World Health Organization and Project ECHO® collecting and processing your personal data including your name, institutional affiliation, email address in accordance with WHO’s personal data protection framework for the purpose of event registration and managing attendance, event follow-up, contacting you about future events, and preparing statistics of global event participation by region. Please note that a recording of the event may be made publicly available, for example on the WHO website.

* mandatory question

^#^ data collected from February 2022 onwards
