# Supplementary material for: WHO public health laboratories webinar series – an online platform to disseminate testing recommendations and best practices during health emergencies
Source: Front Public Health. 2025 Jan 15;12:1462756. doi: 10.3389/fpubh.2024.1462756 (PMC11775005; doi:10.3389/fpubh.2024.1462756)
Supplement: Supplementary file 3 [file Table_3.docx]

Supplementary Material

**Supplementary Material 3. Annual survey questionnaire**

Thank you for participating in the WHO Public Health Laboratories knowledge sharing webinar series!

You are invited to complete this survey because you participated in this webinar series at least once in the past 12 months. As we look back over the past year, we want to know what has been most useful and how these webinars could be improved, as well as how you have applied what you learned and what you want to learn more about. We did a similar survey last year and used results to make improvements and prioritize discussion and learning topics.

This survey will take about 10 minutes. Your responses are anonymous – it does not require your name, email, or other identifying information. You can choose to share your email at the end of the survey, but it will not be reported with your responses.

Thank you for your time and feedback!

1. How often did you attend the WHO Public Health Laboratories sessions in the past year?

❑ Attended more than 10 sessions

❑ Attended between 6 and 10 sessions

❑ Attended between 1 and 5 sessions

❑ Registered and did not attend

❑ Did not register or attend

1. Which of these were barriers to attending, if any? Check all that apply.

❑ Time of day

❑ Length of sessions

❑ Language

❑ Software (e.g., Zoom)

❑ Internet connection

❑ Could not find log-in information when needed

❑ Session topic not relevant to me

❑ Other (please describe briefly)

❑ None of the above

1. How many WHO Public Health Laboratories session recordings did you watch in the past year?

❑ Watched more than 10 session recordings

❑ Watched 6 to 10 session recordings

❑ Watched 1 to 5 session recordings

❑ Did not watch any recordings

1. How helpful were each of these elements in this webinar series?

|  | **Not very helpful** | **Somewhat helpful** | **Very**  **helpful** |
| --- | --- | --- | --- |
| Hearing experiences from other countries |  |  |  |
| Learning from peers |  |  |  |
| Learning latest technical developments |  |  |  |
| Direct contact with WHO |  |  |  |
| Links to WHO documents |  |  |  |
| Technical education (e.g., sequencing) |  |  |  |
| Direct contact with colleagues in other countries |  |  |  |
| Direct contact with colleagues in similar roles to mine |  |  |  |
| Other (Please describe: _____________) |  |  |  |

1. Have you learnt new knowledge or skills in one or more of the areas covered? Check all that apply.

❑ Quality Management System

❑ Biosafety and biosecurity

❑ Emergency Management and Response

❑ Workforce Training

❑ General Laboratory Practice and testing methods

❑ Laboratory information systems

❑ Surveillance

❑ Bioinformatics

❑ Research

❑ Ethics

❑ Management and Leadership

❑ Communication

❑ Other (Please describe: _________________)

❑ None of the above

1. How did you use what you learned in these sessions, if at all? Check all that apply.

❑ Used general information

❑ Used specific documents and links

❑ Looked up additional information

❑ Shared with colleagues

❑ Changed laboratory procedures or practices

❑ Changed guidelines, protocols, or policies

❑ Other (Please describe: ____________________)

❑ None of the above

1. Please briefly describe how you applied what you learned.

1. Which of these were barriers to using what you learned? Check all that apply.

❑ No opportunities to apply in my work

❑ Need more training

❑ Lack of support from supervisor

❑ Lack of support from co-workers

❑ Lack of time

❑ Lack of resources

❑ Differing guidance from my government

❑ Other (Please describe: ____________________)

❑ None

1. How likely are you to recommend this webinar series to a colleague?

| Not at all  likely | |  | |  | |  | |  | |  | |  | | Extremely  likely | | |
| --- | --- | --- | --- | --- | --- | --- | --- | --- | --- | --- | --- | --- | --- | --- | --- | --- |
| 1 | 2 | | 3 | | 4 | | 5 | | 6 | | 7 | | 8 | | 9 | 10 |
|  |  | |  | |  | |  | |  | |  | |  | |  |  |
|  |  |  |  |  |  |  |  |  |  |  |  |  |  |  |  |  |

10. How could the session format improve the balance between lecture and interactivity?

❑ Less lecture and more interactivity, with smaller group discussions or workshops

❑ Less interactivity and more lecture

❑ No changes, right amount of lecture and interactivity learning

❑ Other (Please describe: ____________________)

11. What other recommendations do you have for improving this webinar series?

1. In which WHO region are you?

❑ African

❑ Eastern Mediterranean

❑ European

❑ Americas

❑ South-East Asia

❑ Western Pacific

1. Which of these best describes your role?

❑ Laboratory personnel

❑ Medical care provider

❑ Programme manager

❑ Technical officer

❑ Consultant

❑ Public health official

❑ Researcher

❑ Student

❑ Other (Please describe: ______________________________)

We would like to follow up with a few individuals for more in-depth feedback. Please share your email address if you are willing to be contacted, and we may contact you.

[Email field]

Thank you for your feedback!
