# Supplementary material for: WHO public health laboratories webinar series – an online platform to disseminate testing recommendations and best practices during health emergencies
Source: Front Public Health. 2025 Jan 15;12:1462756. doi: 10.3389/fpubh.2024.1462756 (PMC11775005; doi:10.3389/fpubh.2024.1462756)
Supplement: Supplementary file 4 [file Table_4.docx]

Supplementary Material

**Supplementary Material 4. Focus group discussions semi-structured guide**

- Let’s beginning with learning a little about your interests. Can you briefly share why you chose to participate in this webinar series?
- Thinking back about the sessions you attended, can you briefly share which one you enjoyed the most and why? (share in the chat the session titles)
  - Possible probes
    - Does anybody else want to share something they enjoyed?
    - Did anyone else feel the same way during that session?
- Thinking back to the webinar topics, what is something you were able to change in your work due to these sessions?
  - Possible probes
    - List of webinar topics in the chat
    - Does anybody else want to share a change you made in your work?
    - Can you tell us more about how this series led to that change?
    - Does anybody else want to share something you learned during the series that prompted you to make a change in your work?
- Sometimes there are barriers to making changes in your work - was there anything you wanted to change based on this series but there were barriers to doing so?
  - Possible probes
    - Does anybody else have another experience of difficulty in making changes to their work but saw the potential?
    - Can you elaborate a bit more on what the barriers were?
    - Did anyone have similar or different experiences of change in your work you would like to share?
- How about elements of the series you didn't enjoy or think could be improved? Was there anything about the sessions' content, presentation, or organization you would recommend changing?
  - Possible probes
    - Topics
    - Time/length
    - Frequency
    - Format
    - Discussion
- Is there anything else you would like to share about the Public Health Laboratories webinar series that we haven’t covered?
