# Supplementary material for: WHO public health laboratories webinar series – an online platform to disseminate testing recommendations and best practices during health emergencies
Source: Front Public Health. 2025 Jan 15;12:1462756. doi: 10.3389/fpubh.2024.1462756 (PMC11775005; doi:10.3389/fpubh.2024.1462756)
Supplement: Supplementary file 5 [file Table_5.docx]

Supplementary Material

**Supplementary Material 5. Definitions of knowledge application themes**

- Implemented procedures based on risk assessment
  - This theme includes quotes that mention learnings about risk assessment in a laboratory context, with explicit examples of changes in the participant's setting or practice, for instance, how to rearrange the workplace and work schedules according to levels of perceived risk.
- Increased awareness and understanding of potential risks
  - This theme includes quotes that mention learnings about risk assessment in a laboratory context, without explicit examples of changes in the participant's setting or practice, but emphasizing better understanding or awareness of specific risks.
- Training
  - This theme includes quotes that mention how the webinar series prompted participants to design activities for settings of great need as perceived by them or update local training based on the newly acquired knowledge.
- Shared with colleagues
  - This theme includes quotes about sharing the knowledge acquired during the webinar series with colleagues.
- Improved laboratory management skills
  - This theme includes specific quotes about changes in laboratory management skills with inferences or affirmations about its beneficial consequences.
- Informed national protocols
  - This theme includes quotes about how the series informed updates made to national protocols or how a participant was able to advise the Ministry of Health in creating country-specific guidelines.
- Adapted to other contexts
  - This theme includes one quote about how the knowledge acquired was critical and could be adapted to different contexts, in this case, in response to an Ebola outbreak.
